# Uptake of hepatitis B vaccination and associated factors among health sciences students, Mogadishu, Somalia

**DOI:** 10.3389/fpubh.2023.1203519

**Published:** 2023-09-14

**Authors:** Yahye Sheikh Abdulle Hassan, Shafie Abdulkadir Hassan, Nur Rashiid Ahmed

**Affiliations:** ^1^Department of Nursing and Midwifery, Faculty of Medicine and Health Sciences, Jamhuriya University of Science and Technology, Mogadishu, Somalia; ^2^Department of Medical Microbiology, Faculty of Medical Laboratory Sciences, University of El imam El Mahdi, Kosti, Sudan; ^3^Department of Medical Laboratory Sciences, Faculty of Medicine and Health Sciences, Jamhuriya University of Science and Technology, Mogadishu, Somalia; ^4^Advance Medical Research Centre, Jamhuriya University of Science and Technology, Mogadishu, Somalia

**Keywords:** HBV, knowledge, attitude, practice, vaccination, students

## Abstract

**Background:**

Hepatitis B is a potentially fatal liver infection caused by the hepatitis B virus (HBV). It is a serious issue for global health. It considerably raises the risk of cirrhosis and liver cancer-related death and can result in chronic infection. The risk of infection is high among health sciences students due to the risk of occupational contact with fluids of infected patients and the risk of needle stick injury. The most effective way of preventing HBV infection is the vaccination of students prior to their posting to healthcare settings. There is no data available about HBV vaccination uptake among Health Sciences students in Somalia. Therefore, this study aimed to determine HBV vaccination uptake and associated factors toward HBV among health science students in Somalia.

**Methods:**

A cross-sectional study was undertaken among health sciences students from August to October 2022. Data were gathered using Kobo Toolbox using a standardized questionnaire with questions on characteristics, knowledge attitude, and HBV prevention practices. A total of 569 students were involved in the study. Stata version 15 was utilized for the analysis. Bivariate and multivariate logistic regression analysis, as well as descriptive statistics, were performed. In order to assess the existence and significance of the relationship between the outcome and risk factors, an adjusted odds ratio with a 95% confidence interval (CI) was used. Statistical significance was considered as a *p*-value ≤0.05.

**Results:**

Of the 569 study participants, 33.4% (95%CI: 29.6–37.4) received a full dose of the HBV vaccine in this study. Participants had good HBV prevention knowledge, attitudes, and practices at 69.6, 37.96, and 50.6%, respectively. The lack of access and the high cost of the vaccine were the reasons for not taking the vaccine. Second-year [AOR: 0.22 (0.12–0.43)]. Positive attitude [AOR: 0.54 (0.31–0.93)], and good practice [AOR: 6.99 (3.62–13.5)].

**Discussion:**

The study indicated that 33.4% of health sciences students had received the required HBV vaccination doses, academic year, attitude, and practice were significantly associated with full-dose vaccination status. The unavailability of the vaccine and the high cost of vaccination were the most common reasons for not taking the vaccine. It is recommended that students receive vaccinations before beginning clinical rotations, and give instruction on infection prevention strategies and general precautions, particularly regarding HBV infection.

## Background

Worldwide, hepatitis B virus (HBV) infection is a serious public health issue. Acute infections and chronic conditions, including hepatitis, cirrhosis, and hepatocellular carcinoma, account for a significant portion of the global burden of disease and mortality (1). The World Health Organization (WHO) estimates that two billion people have current or past HBV infection symptoms, and an additional 257 million people have a chronic infection and are at risk of developing HBV-related liver disease (2, 3). The virus may spread through hospitals as a result of contact with contaminated body fluids, blood, blood products, needlestick wounds, or sharp objects (such as scalpels or needles) used on infected patients (4). As Somalia tries to get back on its feet after a terrible civil war, hepatitis virus infections are a major health risk. As a result, comprehensive national initiatives and recommendations for the care of individuals with hepatitis have been established (5). According to the World Health Organization, the prevalence of hepatitis B virus infection is especially high in Somalia (>8) (6). Health science students are more likely to contract HBV at work because of frequent occupational exposure to blood and bodily fluids during clinical rotations (7). Results from several epidemiological studies indicate varying rates of HBV prevalence among medical and health science students, including 4.6, 11, and 9% at the University of Lome, Togo (8), and Ethiopia (9), respectively. The World Health Organization (WHO) has reported that hepatitis B vaccination coverage among healthcare workers (HCWs) in developing countries is estimated to be within the range of 18–39%, while in developed countries the range is between 67–79% (2). Health science students, like other healthcare workers, are equally at risk of exposure to infectious agents and hazardous materials when they come into contact with patients and contaminated instruments during their professional practice.

Only 16.4% of HCWs were found to have received all recommended doses of HBV vaccination in a study conducted in Bosaso, Puntland, and Somalia (10). Similarly, another study carried out among health science students in Ethiopia showed that only 5.8% of students were fully vaccinated (11). The main reasons for not receiving the vaccine included unavailability of the vaccine, high cost, lack of time, and fear of vaccine side effects (10, 11). Currently, there is no study available that has determined the HBV vaccination status and associated factors among health science students in Somalia who are on clinical placement. In order to create efficient methods for HBV prevention and control, it is necessary to comprehend the vaccination coverage within this group of healthcare personnel. It’s also worth noting that the WHO has a goal of completely eliminating HBV by the year 2030 (12).

Vaccination is generally agreed upon as the most effective and practical method of preventing hepatitis B virus infection (13). For those in high-risk professions and academic programs, the World Health Organization (WHO) recommends vaccination (14). Students in the health sciences should get immunized before beginning clinical rotations to help minimize the spread of disease and protect themselves from getting sick during their rotations. There is not much information on HBV vaccination status and associated factors among health science students in Somalia, despite the country’s rising HBV infection rate. Consequently, the aim of this was to assess the uptake of HBV vaccination and associated factors among health science students in Mogadishu.

## Methods

### Study design

This study employed a cross-sectional online survey to ascertain the uptake of hepatitis B vaccination and associated factors among health sciences students in Mogadishu, Somalia. The research was undertaken for 3 months, from July to September 2022, and involved 569 students.

### Sample size determination and sampling technique

An online sample size calculator was used to determine an appropriate sample size for a study with a 3% margin of error and a 95% confidence interval. The target population was made up of 1,215 students. 50% since the Proportion of HBV vaccination status among health sciences students was unknown. As a result, the final sample size for the study for this study was 569. The population was sampled using purposive nonprobability sampling technique.

### Data collection tool

Participants’ information was collected using a structured questionnaire. The application was distributed *via* Kobo Toolbox. The questionnaire was previously utilized and validated in an Ethiopian study (11). The questionnaire is divided into four sections, with section one containing questions about demographic information (gender, age in year, department, and academic year). The second knowledge section contains ten items. If a respondent answered at least 70 percent of knowledge questions correctly, their knowledge was categorized as “good knowledge.” “Poor knowledge” exists if less than 70% of knowledge item questions are correctly answered. Section three contained six items for attitude, which were classified as follows: Positive attitude: if respondents gave the correct response to at least 70 percent of attitude items, Negative attitude: if respondents answered fewer than 70 percent of attitude questions, Section 4 is composed of eight practice questions. Good practice” occurs when study participants are able to correctly answer at least 70% of the practice questions. When participants were unable to answer 70 percent of practice questions correctly. The dependent variable was vaccination status against the hepatitis B virus, while the independent variables were age, gender, residence, department, academic years, and knowledge, attitudes, and practices. The HBV vaccination status variable was encoded as full-dose vaccination, not fully vaccinated, or unvaccinated. Nonetheless, a final analysis was conducted for the status of full-dose vaccination, which has a binary result (yes/no). Prior to participant enrollment, informed consent was obtained.

### Data analysis

After making sure the questions were complete, consistent, and clear, version 15 of STATA was used to look at the data. The primary outcome variable of interest was the vaccination status and reasons for non-vaccination of respondents. For qualitative variables, descriptive statistics like percentage (%) and frequency were used, while the mean and standard deviation were used for quantitative variables. Using multiple logistic regression, the relationship between whether or not a full-dose vaccine had been given and other factors were looked into. Odds ratios were implemented as measures of the association’s strength. A value of *p* of 0.05 or less was considered statistically significant.

## Results

### Sociodemographic characteristics of health sciences students (*n* = 569)

In this study, a total of 569 participants were included. The majority of participants were female (70.47%) and older than 20 years old (68.7%), with a mean age of 21.4 **±** 2.45. The most represented departments were MLS and Nursing, at 29.7 and 28.5%, respectively. In terms of the academic year, the majority of participants (274, or 48.15%) were in their fourth year of study, as shown in Table 1.

**Table 1:** Sociodemographic characteristics of health sciences students.

Variable	Category	Freq.	Percent
Gender	Male	168	29.53%
	Female	401	70.47%
Age category	Less than 20 years	218	38.3%
	Greater than 20 years	351	68.7%
	Mean ± SD 21.4 ± 2.45		
Department	Medical Laboratory Sciences	169	29.70%
	Nursing	162	28.5%
	PH	143	25.13%
	Midwifery	95	16.70%
Academic year	First	37	6.50%
	Second	119	20.91%
	Third	139	24.43%
	Fourth	274	48.15%
Total		569	100.00

MLS, Medical Laboratory Sciences; PH, Public Health.

### Knowledge of health sciences students toward hepatitis B prevention

The results showed that 72.1% of students had good knowledge of HBV prevention measures, while 27.9% had poor knowledge. Regarding specific knowledge items, 89.1% of students knew that HBV can cause liver cancer, while 84.9% knew that carriers can transmit the infection to others. Only 34.8% of students knew that HBV cannot be transmitted through casual contact such as holding hands, while 77.7% knew that HBV can be spread through contact with open wounds or cuts. Also, 91.9% of students knew that HBV can spread through blood and blood products that are contaminated as described in Table 2.

**Table 2:** Knowledge of health sciences students toward hepatitis B prevention.

Item	Yes	No
Can the hepatitis B virus cause liver cancer?	507 (89.1%)	62 (10.9%)
Can HBV carriers transmit the infection to others?	483 (84.9%)	86 (15.1%)
Can hepatitis B be caught through casual contacts, such as holding hands?	198 (34.8%)	371 (65.2%)
Can hepatitis B be spread through contact with open wounds/cuts?	443 (77.7%)	126 (22.1%)
Can hepatitis B be transmitted by contaminated blood and blood products?	523 (91.9%)	46 (8.1%)
Can hepatitis B be transmitted by un-sterilized syringes, needles, and surgical equipment	517 (90.9%)	52 (9.1%)
Can hepatitis B be cured or treated	260 (45.7%)	309 (54.3%)
Can the HBV vaccine prevent hepatitis B?	519 (91.2%)	50 (8.8%)
Do you think HBV has laboratory tests?	507 (89.1%)	62 (10.9%)
Do you think that HBV has post-exposure prophylaxis?	401 (70.5%)	168 (29.5%)

### Attitude of health sciences students toward hepatitis B prevention

The results in Table 3 demonstrated that 67.7% of students had a positive attitude toward HBV prevention, while 32.3% had a negative attitude. Concerning particular attitude items, 47.5% of students believed that they were not at risk of getting HBV, while 52.6% disagreed with this statement. Additionally, 25.1% of students did not believe in the HBV vaccine, while 74.9% disagreed with this statement. Furthermore, 53.6% of students believed that changing gloves during blood collection and testing was a waste of time, while 46.4% disagreed with this statement. But most of the students (85.4%) agreed that all patients should be tested for HBV before they get medical care, while 14.6% disagreed.

**Table 3:** Attitude of health sciences students toward hepatitis B prevention.

Item	Agree	Disagree
I am not at risk of getting hepatitis B	270 (47.5%)	299 (52.6%)
I do not believe in the hepatitis B vaccine	143 (25.1%)	426 (74.9%)
Changing gloves during blood collection and testing is a waste of time	305 (53.6%)	264 (46.4%)
All patients should be tested for HBV before they receive healthcare	486 (85.4%)	83 (14.6%)
I do not like treating people with HBV	229 (40.3%)	340 (59.6%)
Following infection control guidelines will protect me from being infected with HBV at work.	470 (82.6%)	99 (17.4%)

### Practice of health sciences students toward hepatitis B prevention

The results showed that 76.6% of students had good practice in HBV infection prevention, while 23.4% had negative practice. With respect to particular items, 66.6% of students had undergone screening for HBV, while 33.4% had not. Additionally, 63.1% of students had received the HBV vaccine, while 36.9% had not. Furthermore, 89.6% of students reported always changing gloves for each patient during blood taking, while 10.4% reported not always changing gloves. Similarly, 83.7% of students reported always asking for a new syringe before use, while 16.3% reported not always doing so. In terms of needlestick injuries, 23.4% of students reported having had a needlestick injury. Of those who reported having had a needlestick injury, 92.5% reported the injury to their supervisor or occupational health department as shown in Table 4.

**Table 4:** Practice of health sciences students toward hepatitis B prevention.

Item	Yes	No
Have you done the screening for hepatitis B?	379 (66.6%)	190 (33.4%)
Have you got yourself vaccinated against hepatitis B?	359 (63.1%)	210 (36.9%)
If your answer is yes to question #2, how many doses of hepatitis B vaccine did	One dose	84 (14.8%)
	Two doses	85 (14.9%)
	Three doses	190 (33.4%)
I always change gloves for each patient during blood taking.	510 (89.6%)	59 (10.4%)
Do you ask for a new syringe before use?	476 (83.7%)	93 (16.3%)
Have you ever had a needle prick injury?	322 (56.6%)	247 (43.4%)
I always report for needle stick injury	423 (74.3%)	146 (25.7%)

### Hepatitis B vaccination status among health sciences students

The result of the study revealed that 359 (63.1%) of the sample population had already received vaccination against hepatitis B. Among those who had been vaccinated, 84 (14.8%) received one dose of the vaccine, 85 (14.9%) received two doses, and the majority 190 (33.4%; 95CI 29.5–37.4) received the recommended three doses of the vaccine. However, 36.9% (210 individuals) of the participants reported that they had not been vaccinated against hepatitis B. The most common reason cited for not taking the vaccine was the unavailability of the vaccine (46.2%), high cost of vaccination (26.2%), lack of knowledge about the vaccine (21.4%) and fear of side effects of the vaccine (6.2%).

### Factors associated with full dose vaccination among health sciences students

As shown in Table 5, the study utilized multivariate logistic regressions to test the association between full-dose vaccination status and independent variables. By considering *p*-value <0.25 in bivariate analysis, gender, age, residence, academic year, department, attitude, and practice were identified as candidate variables for multivariate analysis. Following the identification of candidate variables for multivariate analysis, a logistic regression analysis was performed to adjust for potential confounding variables and determine the variables that were significantly associated with full-dose vaccination status. The results of the multivariate analysis indicated that academic year, positive attitude, and practices were found to be significantly associated with full-dose vaccination status.

**Table 5:** Factors associated with full dose vaccination among health sciences students.

Variable	Category	Full-dose of vaccination		AOR (95% CI)
		Yes	No	
Age	≤ 20	61	157	0.91 (0.57–1.46)
	>20	129	222	1
Sex	Male	44	124	1
	Female	146	255	1.49 (0.91–2.83)
Department	MLS	52	117	1
	Nursing	66	96	1.3 (0.77–2.19)
	PH	38	105	0.93.(0.54–1.61)
	Midwifery	34	61	0.86 (0.47–1.59)
Academic year	First-year	8	29	0.42 (0.17–1.06)
	Second year	16	103	0.22 (0.12–0.43)
	Third year	47	92	0.72 (0.44–1.76)
	Fourth-year	119	155	1
Attitude level	Positive attitude	157	336	0.54 (0.31–0.93)
	Negative attitude	33	43	1
Practice	Good	179	257	7.17 (3.71–13.82)
	Poor	11	122	1

MLS, Medical Laboratory Sciences; PH, Public Health; AOR, Adjusted Odd Ratio.

In terms of the academic year, second-year students were much less likely to be vaccinated than fourth-year students, with an AOR of 0.22 (95% CI: 0.12–0.43). This means that second-year students were 78% less likely to be vaccinated compared to fourth-year students.

Regarding attitude level, students with a positive attitude toward vaccination were about 46% less likely to be fully vaccinated compared to those with a negative attitude, with an AOR of 0.54 (95% CI: 0.31–0.93). In terms of vaccination practice level, the results showed that students with good vaccination practices were almost 7 times more likely to be fully vaccinated compared to those with poor practices, with an AOR of 6.99 (95% CI: 3.62–13.5).

## Discussion

This study was conducted to assess the uptake of hepatitis B vaccination among health Sciences students in Mogadishu, Somalia. In developing countries, hepatitis B virus infection is a significant public health concern that results in high levels of illness and death (15).

The findings of the study report that only 33%(95% CI: 29.5–37.4) of vaccination status. The hepatitis B vaccination status in the current study is higher compared to the study conducted in Ethiopia (4.7%) (12), Somalia (16.4%) (10), Cameron 16.81% (16) and Kenya (20.2%) (17). While it is lower than the study carried out in India 57% (18), Uganda 44.3% (19), Nigeria 39.2% (20) and Ethiopia 43.4% (21). The difference could be due to variations in vaccination policy, awareness campaigns, sociocultural factors, the standard of healthcare services, and sample size. It is, however, consistent with other similar studies conducted in Nepal 37% (22). According to study result, the full dose vaccination rate among health sciences students in Somalia was 33%, which falls within the range of 18–39% reported by the WHO for developing countries (2).

The study found 33.4% of health sciences students received a full dose of HBV vaccine, a higher rate than a previous study in Bosaso (23). Factors like implementation variations, healthcare infrastructure, resources, and vaccine access may contribute to these disparities.

The finding of the study reports that only 33% of study participants received a full dose of vaccination against hepatitis B. The most common reason reported for not taking the vaccine was the unavailability of the vaccine (46.2%), high cost of vaccination (26.2%). This finding was in agreement with studies conducted (10, 11, 17, 22). With the potential risk of occupational exposure, this poor coverage reflects a low level of protection against hepatitis B infection, which necessitates policymakers’ intervention.

In term of academic year, there is a statistically significant difference in the likelihood of second-year students being fully vaccinated compared to fourth-year students. Second-year students are less likely to be fully vaccinated, which is consistent with the findings of a study conducted in Ethiopia (11), Uganda (19).

Regarding attitude level, students with a positive attitude toward vaccination were about 46% less likely to be fully vaccinated compared to those with a negative attitude, with an AOR of 0.54 (95% CI: 0.31–0.93). This finding is line with study in Kenya (17). In terms of vaccination practice level, the results showed that students with good vaccination practices were almost 7 times more likely to be fully vaccinated compared to those with poor practices, with an AOR of 6.99 (95% CI: 3.62–13.5). This is similar to the finding of a previous study that showed that practice level is significantly associated with full dose vaccination (11).

This study has a few significant limitations. Firstly, the study’s cross-sectional design does not support a relationship between cause and effect. Lastly, this study may also be limited by the self-reported nature of the data, which may have introduced bias or inaccuracies into the results.

## Conclusion

The study shows that only 33% of participants received a full vaccination against hepatitis B. Academic year, attitude, and practice were significantly associated with full vaccination. The unavailability of the vaccine and the high cost of vaccination were the most common reasons for not taking the vaccine. The study reveals that students who are in clinical training are very likely to get HBV because they do not get vaccinated. So, the study suggests that all healthcare students get vaccinated before they start their clinical placements, receive training on infection prevention techniques and universal precautions specifically for HBV, and raise awareness on attitudes that improve the uptake of the hepatitis B vaccination. Also, the study suggests that efforts should be made to ensure that the vaccine is more widely available and accessible to individuals who need it and that cost-reduction strategies should be implemented to ensure that students are not deterred from obtaining the vaccine due to financial constraints.

## Data availability statement

The datasets presented in this study can be found in online repositories. The names of the repository/repositories and accession number(s) can be found in the article/supplementary material.

## Ethics statement

Ethical approval for this study was obtained from the Ethics and Research Committee of Jamhuriya University of Science and Technology, Mogadishu, Somalia. The approval reference number is (JUREC0048/FMHS0027/052022). Before participating in the survey, all the participants provided their informed consent for inclusion. The current study’s methods were carried out in compliance with appropriate guidelines and regulations.

## Author contributions

YH played an integral role in conceptualizing the research, developing the methodology, conducting formal analyses, investigating the results, and writing the original draft. SH contributed to the research by developing the methodology, conducting formal analyses, investigating the results, and reviewing and editing the manuscript. NA played an important role in conceptualizing the research, developing the methodology, reviewing and editing the manuscript, supervising the project, and acquiring the necessary funding to complete the study. All authors have reviewed and approved the final version of the manuscript.
